# Zika Virus NS2A-Mediated Virion Assembly

**DOI:** 10.1128/mBio.02375-19

**Published:** 2019-10-29

**Authors:** Xianwen Zhang, Xuping Xie, Hongjie Xia, Jing Zou, Linfen Huang, Vsevolod L. Popov, Xinwen Chen, Pei-Yong Shi

**Affiliations:** a Department of Biochemistry & Molecular Biology, University of Texas Medical Branch, Galveston, Texas, USA; b State Key Laboratory of Virology, Wuhan Institute of Virology, Chinese Academy of Sciences, Wuhan, People’s Republic of China; c University of Chinese Academy of Sciences, Beijing, People’s Republic of China; d Department of Pathology, University of Texas Medical Branch, Galveston, Texas, USA; e Institute for Human Infections & Immunity, University of Texas Medical Branch, Galveston, Texas, USA; f Guangzhou Institute of Biomedicine and Health, Chinese Academy of Sciences, Guangzhou, People’s Republic of China; g Sealy Institute for Vaccine Sciences, University of Texas Medical Branch, Galveston, Texas, USA; h Sealy Center for Structural Biology & Molecular Biophysics, University of Texas Medical Branch, Galveston, Texas, USA; i Department of Pharmacology & Toxicology, University of Texas Medical Branch, Galveston, Texas, USA; Duke University Medical Center

**Keywords:** Zika, flavivirus, virus assembly

## Abstract

The flavivirus virion consists of an envelope outer layer, formed by envelope (E) and membrane (M) proteins on a lipid bilayer, and an internal core, formed by capsid (C) protein and genomic RNA. The molecular mechanism of flavivirus assembly is not well understood. Here, we show that Zika virus (ZIKV) NS2A protein recruits genomic RNA, the structural protein prM/E complex, and the NS2B/NS3 protease complex to the virion assembly site and orchestrates virus morphogenesis. Coimmunoprecipitation analysis showed that ZIKV NS2A binds to prM, E, NS2B, and NS3 (but not C, NS4B, or NS5) in a viral RNA-independent manner, whereas prM/E complex does not interact with NS2B/NS3 complex. Remarkably, a single-amino-acid mutation (E103A) of NS2A impairs its binding to prM/E and NS2B/NS3 and abolishes virus production, demonstrating the indispensable role of NS2A/prM/E and NS2A/NS2B/NS3 interactions in virion assembly. In addition, RNA-protein pulldown analysis identified a stem-loop RNA from the 3ʹ untranslated region (UTR) of the viral genome as an “RNA recruitment signal” for ZIKV assembly. The 3ʹ UTR RNA binds to a cytoplasmic loop of NS2A protein. Mutations of two positively charged residues (R96A and R102A) from the cytoplasmic loop reduce NS2A binding to viral RNA, leading to a complete loss of virion assembly. Collectively, our results support a virion assembly model in which NS2A recruits viral NS2B/NS3 protease and structural C-prM-E polyprotein to the virion assembly site; once the C-prM-E polyprotein has been processed, NS2A presents viral RNA to the structural proteins for virion assembly.

## Abstract (precis)

ZIKV is a recently emerged mosquito-borne flavivirus that can cause devastating congenital Zika syndrome in pregnant women and Guillain-Barré syndrome in adults. The molecular mechanism of ZIKV virion assembly is largely unknown. Here, we report that ZIKV NS2A plays a central role in recruiting viral RNA, structural protein prM/E, and viral NS2B/NS3 protease to the virion assembly site and orchestrating virion morphogenesis. One mutation that impairs these interactions does not significantly affect viral RNA replication but selectively abolishes virion assembly, demonstrating the specific role of these interactions in virus morphogenesis. We also show that the 3ʹ UTR of ZIKV RNA may serve as a “recruitment signal” through binding to NS2A to enter the virion assembly site. Following a coordinated cleavage of C-prM-E at the virion assembly site, NS2A may present the viral RNA to C protein for nucleocapsid formation followed by envelopment with prM/E proteins. The results have provided new insights into flavivirus virion assembly.

## INTRODUCTION

Many flaviviruses are significant human pathogens, including Zika (ZIKV), dengue (DENV), yellow fever (YFV), West Nile (WNV), Japanese encephalitis (JEV), and tick-borne encephalitis (TBEV) viruses. Flaviviruses are formed by 180 copies of envelope (E) and membrane (M) proteins that are anchored onto a host-derived lipid bilayer (1). The viral envelope encloses a nucleocapsid core composed of one copy of genomic RNA and multiple copies of capsid (C) protein. The flavivirus genome is a single-stranded, plus-sense RNA of about 11,000 nucleotides. The viral genome consists of a 5ʹ untranslated region (UTR), a long open reading frame (ORF), and a 3ʹ UTR. The ORF encodes three structural proteins (C, prM, and E) and seven nonstructural proteins (NS1, NS2A, NS2B, NS3, NS4A, NS4B, and NS5). The structural proteins, together with the genomic RNA, assemble into virions. The nonstructural proteins are responsible for viral RNA synthesis, virus morphogenesis, and evasion of the host innate immune response (2).

The molecular mechanism of flavivirus assembly remains largely unknown. Flavivirus and other RNA virus assemblies are coupled with RNA replication (3, 4). During flavivirus assembly, newly synthesized plus-sense RNAs are released from the viral replication complex. The nascent RNA is assembled with C protein into the nucleocapsid core, which is enveloped with prM and E proteins through budding into the endoplasmic reticulum (ER) lumen, resulting in progeny virion. An initial virion, consisting of 60 icosahedrally arranged prM/E heterotrimers, is immature and noninfectious. As the immature virion translocates through the Golgi network, the low-pH environment triggers prM cleavage into pr and M by the cellular furin protease, leading to an infectious, mature virus (5, 6). Since expression of prM/E alone is enough to produce empty virus-like particles, a mechanism has yet to be determined in coupling nucleocapsid formation and subsequent envelopment during virion morphogenesis. Besides structural proteins, several nonstructural proteins, including NS2A, NS2B, and NS3, have been implicated in modulating flavivirus assembly. NS2A protein has distinct roles in flavivirus RNA replication and virion assembly (7–14). Besides functioning as an NS3 protease cofactor, NS2B participates in JEV assembly (15). The helicase domain of NS3 also modulates YFV assembly (16). However, how these nonstructural proteins coordinate genomic RNA and structural proteins to assemble virions remains unknown.

The current study uses ZIKV as a model to study flavivirus assembly. We demonstrate a central role of ZIKV NS2A in recruiting viral RNA, prM/E complex, and NS2B/NS3 complex to virion assembly sites. A 3ʹ UTR stem-loop RNA of the viral genome may serve as a “recruitment signal” to bind to a cytoplasmic loop of NS2A. Mutations that impair the interactions of NS2A with viral RNA, prM/E, or NS2B/NS3 are lethal for virion assembly. These results suggest that ZIKV NS2A recruits viral RNA, unprocessed C-prM-E polyprotein, and NS2B/NS3 protease to the virion assembly site where coordinated cleavage of C-prM-E initiates genomic RNA and C protein encapsidation followed by prM/E envelopment and virion budding.

## RESULTS

### Construction and characterization of a stable HA-NS2A ZIKV.

**FIG 1:**
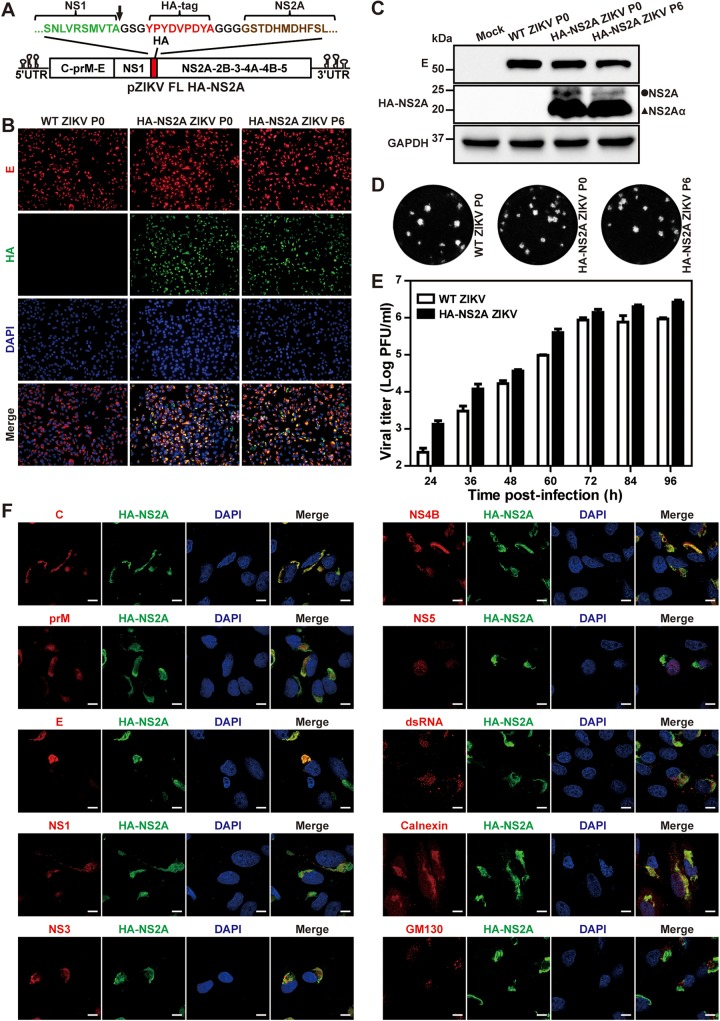
Generation and characterization of HA-NS2A ZIKV. (A) Construction of HA-NS2A ZIKV cDNA clone (pZIKV FL HA-NS2A). The arrow indicates the cleavage site at the C terminus of NS1. (B) IFA of Vero cells infected with WT or HA-NS2A ZIKV. Equal amounts of WT ZIKV and HA-NS2A ZIKV RNAs were electroporated into Vero cells to produce recombinant viruses (P0). The P0 virus was passaged for six rounds (P6) on Vero cells. Immunofluorescence assay was performed on P0 or P6 virus-infected cells (MOI of 0.3) at 48 h postinfection. Viral E protein, HA-NS2A protein, and nuclei were stained with 4G2 antibody (red), HA antibody (green), and 4′,6-diamidino-2-phenylindole (DAPI) (blue), respectively. (C) Western blot analysis of viral E and HA-NS2A in cells infected with WT ZIKV (P0), HA-NS2A ZIKV (P0), or HA-NS2A ZIKV (P6). The cellular GAPDH was used as a loading control. The circle and triangle indicate NS2A and NS2Aα, respectively. (D) Plaque morphologies. Plaques were developed on Vero cells after 4.5 days of infection. (E) Growth kinetics. Vero cells were infected with WT ZIKV and HA-NS2A ZIKV (P0) on Vero cells at an MOI of 0.01. Supernatants were collected at given time points and subjected to plaque assay. Data present the means and standard deviations from three independent experiments. (F) Confocal fluorescence analysis. Infected Vero cells were fixed with 4% paraformaldehyde at 24 h p.i. Viral C, prM, E, NS1, NS3, NS4B, NS5 proteins, and dsRNA were probed with specific antibodies. ER, Golgi apparatus and nuclei were stained with calnexin antibody, GM130 antibody, and Hoechst 33342, respectively. Bar, 10 μm.

One major challenge in studying flavivirus NS2A is the lack of good antibodies against this protein. We failed to raise antibodies for flavivirus NS2A after many attempts using different approaches (data not shown). To overcome this challenge, we constructed a hemagglutinin (HA)-tagged NS2A ZIKV (HA-NS2A ZIKV) using an infectious cDNA clone of Cambodian strain FSS13025 (Fig. 1A). The HA tag was inserted into the junction between NS1 and NS2A genes. Two peptide linkers, with amino acid sequences of “GSG” and “GGG,” were fused to the N and C terminus of the HA tag, respectively. The linkers ensure the correct cleavage of the C terminus of NS1 protein and the flexibility between the HA tag and NS2A protein. The HA-NS2A ZIKV RNA and wild-type (WT) ZIKV RNA (without HA tag) were transfected into Vero cells to generate passage 0 (P0) viruses. To examine the effect of HA tag on viral replication, we compared the viral replications between the P0 HA-NS2A ZIKV and WT ZIKV. Vero cells were infected with the two viruses at a multiplicity of infection (MOI) of 0.3. Immunofluorescence assay (IFA) showed that the numbers of viral E protein-positive cells were comparable between the HA-NS2A ZIKV- and WT ZIKV-infected cells (Fig. 1B). HA-positive cells were detected only in the HA-NS2A ZIKV-infected cells, and the HA staining overlapped the E fluorescent signal (Fig. 1B). When probed with HA antibody, Western blots showed two bands from the HA-NS2A ZIKV-infected cell lysates but not in the WT ZIKV-infected cell lysates (Fig. 1C). The minor band of ∼25 kDa matched the predicted molecular mass of full-length HA-NS2A fusion protein, whereas the smaller major band may represent HA-NS2Aα (representing a C-terminally truncated form of NS2A) as previously reported for YFV (13). The HA-NS2A ZIKV produced plaques equivalent to the WT ZIKV (Fig. 1D). Corroboratively, the replication kinetics of the HA-NS2A ZIKV were also comparable to the WT ZIKV (Fig. 1E). To assess the stability of HA-NS2A ZIKV, we continuously cultured the virus on Vero cells for six rounds (4 days per passage) and characterized the replication of P6 HA-NS2A ZIKV. The P6 HA-NS2A ZIKV exhibited viral replication comparable to the P0 virus, as indicated by IFA (Fig. 1B), Western blotting (Fig. 1C), and plaque morphology (Fig. 1D). Sequencing of the P6 HA-NS2A ZIKV confirmed the retention of the HA tag without any extra mutations. Altogether, the results demonstrate that (i) HA-NS2A ZIKV is stable and (ii) it replicates as well as WT ZIKV.

Using the HA-NS2A ZIKV, we examined the intracellular distribution of NS2A protein in infected cells. IFA was performed on HA-NS2A ZIKV-infected Vero cells using antibodies against HA tag, viral proteins, and cellular markers. The subcellular localization of the proteins was examined under a confocal microscope (Fig. 1F). HA-NS2A displayed a perinuclear distribution pattern that colocalized with calnexin (an ER marker) but not with GM130 (a *cis*-Golgi marker), indicating that NS2A is associated with the ER membrane. The HA-NS2A was also colocalized with viral C, prM, E, NS1, NS3, and NS4B proteins, as well as double-stranded RNA (dsRNA). Because NS5 mainly resided in the cell nuclei, it did not show a strong fluorescent signal in the cytosol to costain with NS2A protein (Fig. 1F). The dominant localization of NS5 in nuclei has been reported for many flaviviruses (17–21). Nevertheless, the results suggest that HA-NS2A ZIKV could be used to study NS2A in viral replication.

### Mutant E103A HA-NS2A ZIKV is defective in virion assembly.

**FIG 2:**
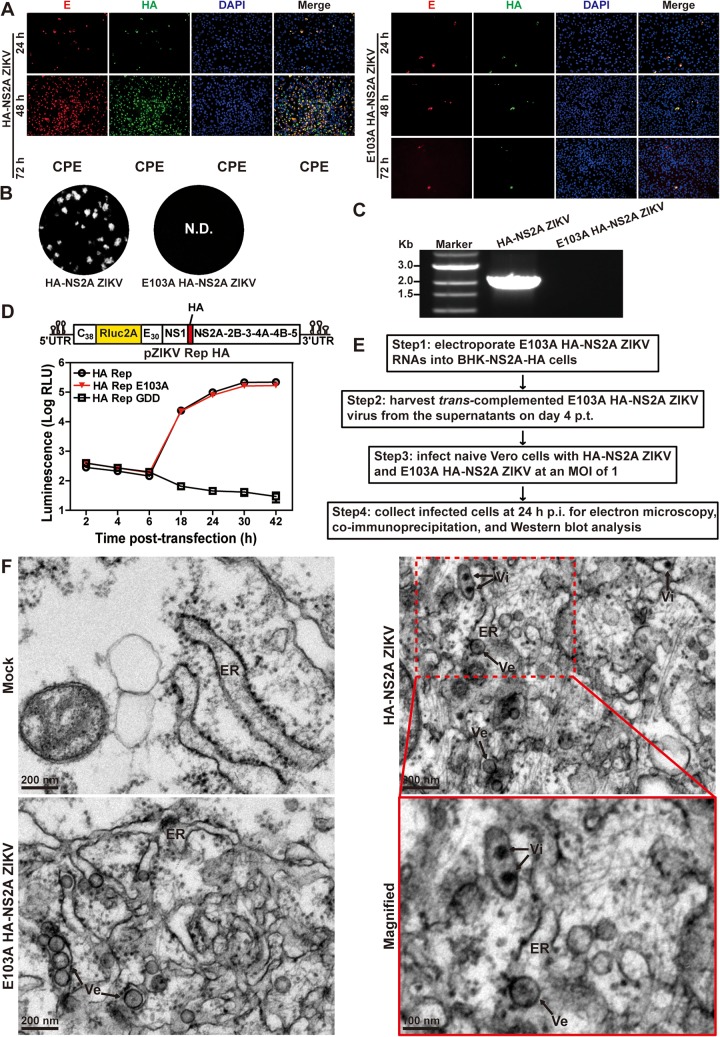
Characterization of the NS2A E103A mutation in HA-NS2A ZIKV. (A) IFA of E and HA-NS2A protein expression in transfected cells. Ten micrograms of HA-NS2A ZIKV and E103A HA-NS2A ZIKV RNAs was electroporated into Vero cells. Cytopathic effect (CPE) was observed in the HA-NS2A ZIKV RNA-transfected cells at 72 h posttransfection. (B) Plaque morphologies of HA-NS2A ZIKV and E103A HA-NS2A ZIKV. N.D., not detected. (C) RT-PCR analysis of extracellular viral RNA. At 24 h p.t., the transfected cells from panel A were washed three times with PBS and maintained in fresh medium. At 120 h p.t., extracellular RNAs were extracted from culture supernatants and examined for viral RNA by RT-PCR. The RT-PCR products were resolved on an 0.8% agarose gel. (D) ZIKV luciferase replicon assay. The top panel depicts the construction of the ZIKV HA-NS2A luciferase replicon. NS2A E103A and NS5ΔGDD (polymerase active site mutations) were engineered into the HA-NS2A ZIKV replicon. Ten micrograms of replicon RNAs was electroporated into Vero cells. The means and standard deviations from three independent experiments are presented. (E) The workflow of *trans*-complementation of E103A HA-NS2A mutant virus in BHK-NS2A-HA cells. (F) Thin-section transmission electron microscopy (TEM) images of HA-NS2A and E103A HA-NS2A ZIKV-infected Vero cells. Vero cells were infected with HA-NS2A ZIKV and E103A HA-NS2A ZIKV (derived from *trans*-complementation as shown in panel E) at an MOI of 1. At 24 h p.i., cells were fixed for TEM analysis. Vi, virus particles; Ve, virus-induced vesicles; ER, endoplasmic reticulum; Mock, uninfected cells.

We recently identified a class of NS2A mutations that selectively abolished virion assembly without affecting viral RNA synthesis (22). The availability of HA-NS2A ZIKV has provided a means to explore the molecular mechanisms of these NS2A mutants in causing virion assembly defects. We introduced one such mutation, E103A, into the HA-NS2A ZIKV and confirmed the defective phenotype in virion assembly (Fig. 2). After transfection into Vero cells, the WT HA-NS2A ZIKV RNA produced an increasing number of E-positive cells and developed cytopathic effects (CPE) at 72 h posttransfection (p.t.) (Fig. 2A). These phenotypes were not observed in the E103A mutant RNA-transfected cells (Fig. 2A), suggesting no spread of viral infection. Infectious virus and viral RNA were detected from the culture supernatants of the WT HA-NS2A ZIKV RNA-transfected cells, whereas no virus or extracellular viral RNAs were produced from the mutant RNA-transfected cells (Fig. 2B and C). To exclude the possibility that the lack of virion production of the mutant was caused by decreased viral RNA replication, we analyzed the mutational effect on viral RNA synthesis using an HA-NS2A luciferase replicon (Fig. 2D). After transfection into Vero cells, the E103A HA-NS2A replicon produced luciferase signals equivalent to the WT HA-NS2A replicon (Fig. 2D), demonstrating that E103A mutation does not affect viral RNA synthesis.

Next, we used transmission electron microscopy (TEM) to examine the cells that had been infected with the E103A HA-NS2A ZIKV. A single-round infectious E103A HA-NS2A ZIKV was prepared by *trans* complementation (Fig. 2E) in which the mutant viral RNA was transfected into a BHK-21 cell line stably expressing WT NS2A-HA protein (22). The resulting E103A HA-NS2A ZIKV was used to infect Vero cells. At 24 h postinfection (p.i.), the cells were processed for TEM analysis (Fig. 2F). Both WT and mutant virus-infected cells developed virus-induced vesicles (Ve) in the rough ER lumen that are presumed to be the viral replication sites (23), whereas no membrane alteration was observed in mock cells. Virus particles were detected only in the WT virus-infected cells (Fig. 2F). The results demonstrate that the E103A mutant could induce ER rearrangement for RNA replication but did not produce virions.

### NS2A selectively interacts with prM, E, NS2B, and NS3 proteins.

**FIG 3:**
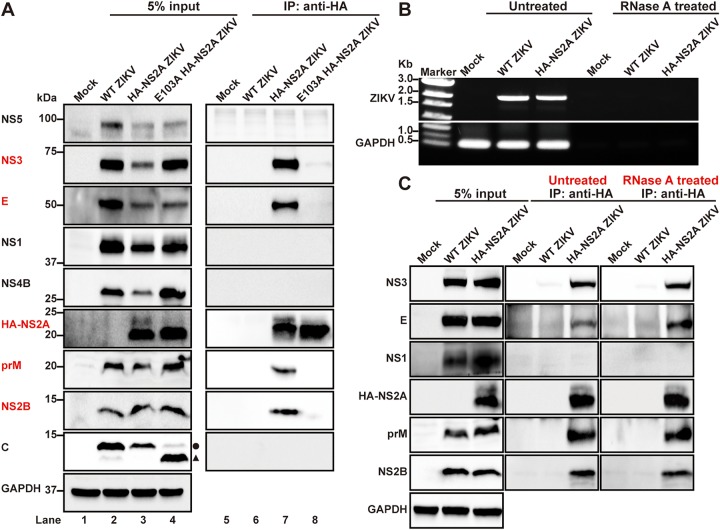
NS2A E103A abolishes the interaction between NS2A and other viral proteins in infected cells. (A) Profiling the interaction of NS2A with other viral protein by co-IP analysis. Vero cells were infected with E103A HA-NS2A ZIKV (derived from *trans*-complementation in Fig. 2E) and HA-NS2A ZIKV at an MOI of 1. At 24 h p.i., coimmunoprecipitation was performed using HA antibody. Viral proteins in both cell lysates (input) and IP eluates were analyzed by Western blotting. The circle and triangle indicate intact C protein and degraded C protein, respectively. (B) RT-PCR analysis of viral RNA in cell lysates before and after RNase A treatment. Vero cells were infected with WT ZIKV (without HA tag) or HA-NS2A ZIKV at an MOI of 0.01. At 72 h p.t., infected cells were harvested and lysed. The cell lysates were then incubated with HA antibody in the presence or absence of RNase A at 4°C overnight. Ninety percent of the mixtures were subjected to co-IP analysis. Ten percent of the cell lysates were used for RNA extraction, followed by RT-PCR to amplify viral RNA or GAPDH mRNA using specific primers. The RT-PCR products were analyzed by an 0.8% agarose gel. (C) Western blot analysis of viral proteins in the input cell lysates and IP eluates from panel B.

Taking advantage of the HA-tagged NS2A protein, we performed coimmunoprecipitation (co-IP) to identify viral proteins that bind to NS2A in ZIKV-infected cells. In WT HA-NS2A ZIKV-infected cells, HA-NS2A selectively pulled down prM, E, NS2B, and NS3 proteins but not C, NS1, NS4B, or NS5 proteins (Fig. 3A, lane 7). We did not test NS4A protein due to the lack of good antibodies against this protein (data not shown). As a negative control, HA antibody did not pull down any viral proteins from the cells infected with the WT ZIKV without HA tag (Fig. 3A, lane 6), demonstrating the specificity of the co-IP result. To examine if the observed HA-NS2A interactions were mediated by viral RNA, we treated the co-IP reaction mixture with RNase A to degrade viral and cellular RNAs. No viral RNA or cellular glyceraldehyde-3-phosphate dehydrogenase (GAPDH) RNA was detected by RT-PCR after the RNase A treatment (Fig. 3B). The RNase A treatment did not affect the HA-NS2A binding to prM, E, NS2B, and NS3 proteins (Fig. 3C), indicating that the observed interactions are independent of RNA.

**FIG 4:**
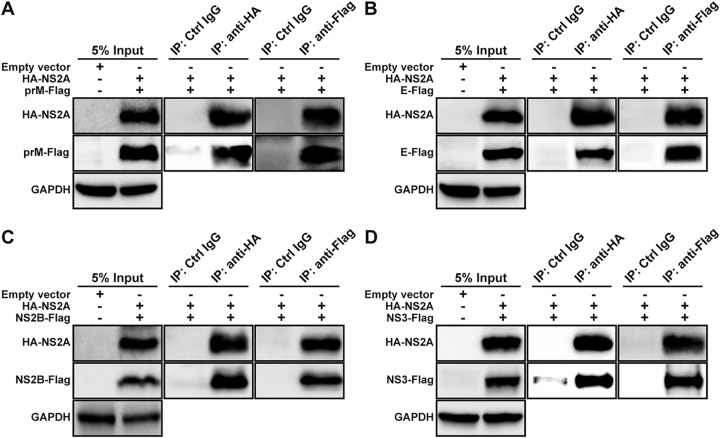
NS2A interacts with prM, E, NS2B, and NS3 proteins in HEK293T cells. HEK293T cells were transfected with two plasmids, one encoding HA-NS2A and the other encoding Flag-tagged prM (A), E (B), NS2B (C), or NS3 (D). At 24 h p.t., cell lysates were precipitated with HA or Flag antibodies. Viral proteins in the cell lysates and IP eluates were detected by Western blotting using corresponding antibodies.

Since prM/E and NS2B/NS3 form individual complexes, we examined if HA-NS2A interacts with one or both proteins from the prM/E and NS2B/NS3 complexes. HEK293T cells were cotransfected with two plasmids: one plasmid encoding HA-NS2A and the other encoding Flag-tagged prM, E, NS2B, or NS3 protein. Co-IP results showed that HA-NS2A pulled down individual prM-Flag (Fig. 4A), E-Flag (Fig. 4B), NS2B-Flag (Fig. 4C), and NS3-Flag (Fig. 4D). These results were further corroborated by reciprocal co-IP data. As negative controls, isotype IgG did not pull down any viral proteins (Fig. 4A to D). These data indicate that ZIKV NS2A can interact with individual prM, E, NS2B, or NS3 protein.

### Mutation E103A abolishes NS2A binding to other viral proteins.

To explore the molecular defect of NS2A E103A on virion assembly, we performed co-IP on cells that were infected with E103A HA-2A ZIKV (prepared through *trans* complementation described in Fig. 2E). Remarkably, E103A HA-NS2A lost its binding to prM, E, NS2B, or NS3 protein (Fig. 3A, lane 8), demonstrating the critical role of these interactions in virion assembly.

**FIG 5:**
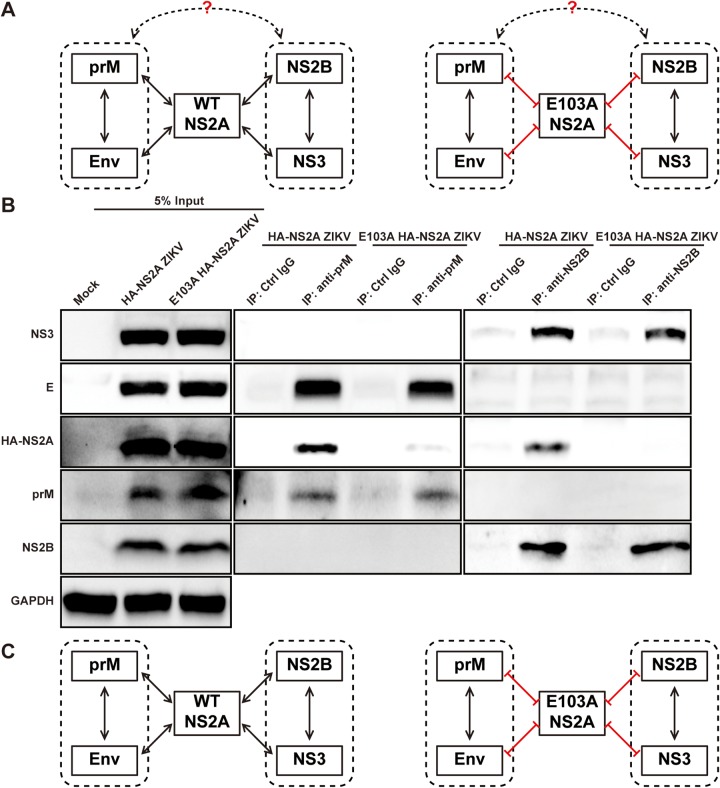
NS2A E103A disrupts the association between prM/E complex and NS2B/NS3 complex. (A) Diagram of the hypothesized interaction network among NS2A, prM/E, and NS2B/3 complexes. Dotted boxes indicate prM/E and NS2B/NS3 complexes. Lines with two arrows indicate protein-protein interactions. Red lines indicate loss of interactions. (B) Co-IP analysis of genomic ZIKV RNA-transfected cells. BHK-21 cells (8 × 10^6^) were electroporated with 2 μg of HA-NS2A ZIKV RNA or 15 μg E103A HA-NS2A ZIKV RNA. The different amounts of RNAs were used to achieve comparable expression levels of viral proteins in HA-NS2A ZIKV- and E103A HA-NS2A ZIKV-transfected cells upon sample collection. Untransfected BHK-21 cells (Mock) were used as control. At 24 h p.t., cells were lysed and subjected to precipitation using prM antibody, NS2B antibody, or isotype control IgG. Viral proteins in the cell lysates and IP eluates were analyzed by Western blotting using corresponding antibodies. (C) Summary of the network among NS2A, prM, E, NS2B, and NS3 proteins. The left panel shows that the WT NS2A acts as an intermediator of prM/E and NS2B/3 complexes. The right panel presents the dissociation between prM/E and NS2B/3 complexes due to the NS2A E103A mutation.

As hypothesized in Fig. 5A (left panel), the prM/E complex could directly interact with NS2B/NS3 complex; alternatively, the prM/E and NS2B/NS3 complexes could indirectly interact through NS2A as an intermediator. The lack of interactions between E103A NS2A and prM/E or NS2B/NS3 enabled us to differentiate between the two possible modes of prM/E and NS2B/NS3 interaction (Fig. 5A, right panel). To address this question, we transfected WT or E103A HA-NS2A ZIKV RNA into BHK-21 cells. At 24 h p.t., cell lysates were immunoprecipitated with antibodies against prM or NS2B. Both prM and NS2B antibodies pulled down WT NS2A in the WT HA-NS2A ZIKV RNA-transfected cells, whereas neither antibody pulled down E103A NS2A in the E103A HA-NS2A ZIKV RNA-transfected cells (Fig. 5B). Importantly, regardless of WT or E103A NS2A, prM antibody pulled down both prM and E proteins but not NS2B or NS3, whereas NS2B antibody pulled down both NS2B and NS3 proteins but not prM or E (Fig. 5B). As a negative control, no viral proteins were pulled down when the samples were precipitated with isotype IgG. These results indicate that, in the context of ZIKV replication, (i) the prM/E complex does not directly interact with the NS2B/NS3 complex and (ii) mutant E103A NS2A does not affect the formation of prM/E or NS2B/NS3 complex.

Taken together, the data support the idea that NS2A serves as an intermediator to interact with the prM/E and NS2B/NS3 complexes (Fig. 5C, left panel). Mutation E103A abolishes NS2A binding to prM/E or NS2B/NS3, leading to the loss of the indirect interaction between prM/E and NS2B/NS3 (Fig. 5C, right panel).

### Capsid cleavage is not responsible for the defective virion assembly of NS2A E103A.

**FIG 6:**
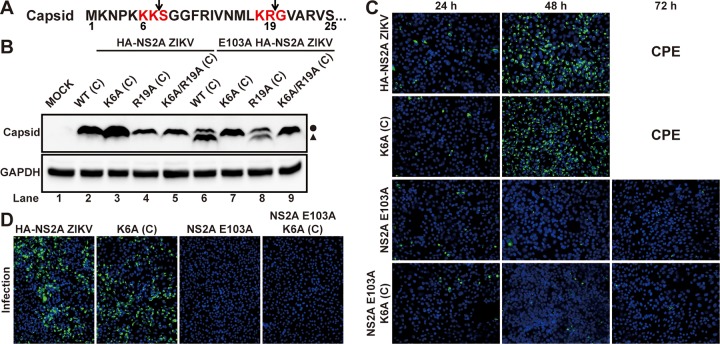
Capsid cleavage is not responsible for the defects of E103A NS2A in virion assembly. (A) Diagram of the two putative viral protease cleavage sites in the first 25 amino acids of C protein. Red residues indicate two potential recognition sequences by viral protease. Arrows indicate the potential viral protease cleavage sites. (B) Western blot analysis of C protein in ZIKV RNA variant-transfected BHK-21 cells. ZIKV RNAs with indicated C mutations in the presence or absence of NS2A E103A were electroporated into BHK-21 cells. At 24 h p.t., cells were collected and lysed. C protein in the lysates was detected by Western blotting using mouse antiserum against capsid. The circle and triangle indicate the intact C protein and cleaved C protein, respectively. (C) IFA of viral E protein expression in the Vero cells transfected with equal amounts of ZIKV RNA variants. (D) IFA of viral E protein expression in infected cells. Vero cells were infected with the culture fluids that were harvested from transfected cells described in panel C. IFA was performed at 24 h p.i.

Besides the loss of the interactions with prM/E and NS2B/NS3 complexes (Fig. 3A, lane 8), the NS2A E103A mutation also resulted in a cleaved C protein (∼13 kDa) in the E103A HA-NS2A ZIKV-infected cells (Fig. 3A, lane 4). Sequence analysis of C protein suggests two potential cleavage sites for viral protease: K_6_K_7_↓S_8_ and K_18_R_19_↓G_20_ (symbol “↓” denotes cleavage site) (Fig. 6A). To determine which cleavage site is responsible for the formation of smaller C, we engineered single- (K6A or R19A) or double-amino-acid mutations (K6A/R19A) to knock out the putative cleavage sites in the WT or E103A HA-HS2A ZIKV RNA. After transfecting the viral RNAs into cells, no cleaved C was detected from the capsid K6A and K6A/R19A (but not R19A) RNAs containing the E103A NS2A mutation (Fig. 6B, lanes 7 to 9), indicating that K_6_K_7_↓S_8_ is responsible for the C cleavage. IFA of the capsid K6A+NS2A E103A viral RNA-transfected cells did not show any increase in E-positive cells from 24 to 72 h p.t. (Fig. 6C). Furthermore, incubation of naive Vero cells with supernatants from the transfected cells did not yield any E-positive cells (Fig. 6D). These results indicate that, despite no C cleavage, the capsid K6A+NS2A E103A viral RNA remains defective in virion production. In contrast, the capsid K6A mutation did not affect the ability of WT viral RNA to produce infectious virus (Fig. 6C and D), excluding the possibility that capsid K6A itself is detrimental to virion assembly. Collectively, these results suggest that capsid cleavage is the consequence, not the cause, of the assembly defect.

### NS2A selectively binds to viral RNA in infected cells.

**FIG 7:**
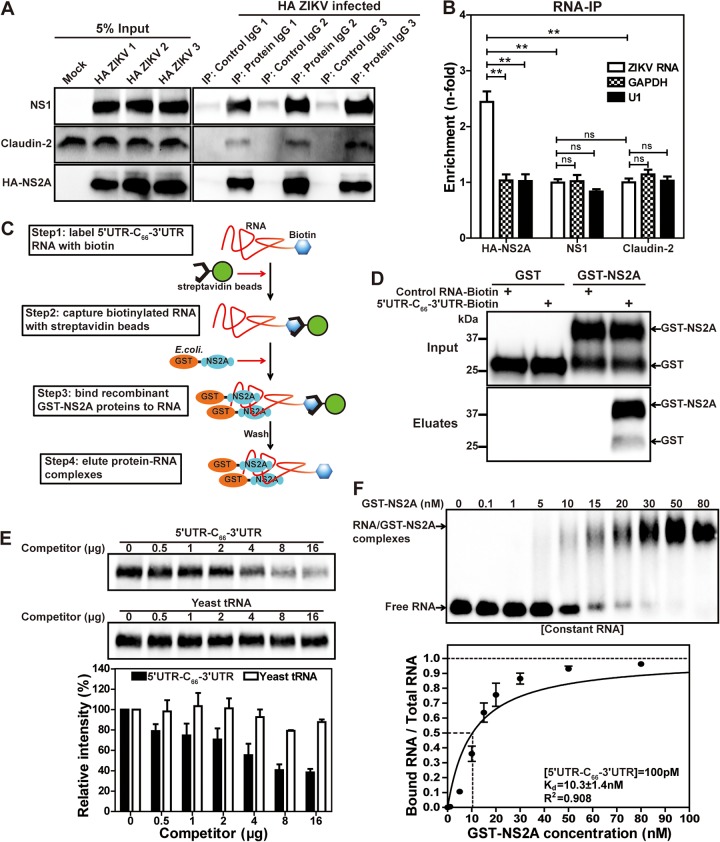
NS2A interacts with viral RNA in ZIKV-infected cells and *in vitro*. (A) Western blot analysis after co-IP. Vero cells were infected with HA-NS2A ZIKV at an MOI of 0.01. At 72 h p.i., cells were collected and lysed. Co-IPs were performed using antibodies against NS1, claudin-2 (control, known to have no RNA binding activities), or HA. An isotype IgG antibody was included as a negative control. NS1, claudin-2, and HA-NS2A in the input cell lysates and IP eluates were analyzed by Western blotting. Three sets of repeating experiments (indicated as 1, 2, and 3) are presented. (B) RT-qPCR analysis of ZIKV or control RNAs after IP. Fifty percent of the IP eluates from panel A were used for RNA extraction, followed by RT-qPCR for detecting viral RNA, cellular GAPDH mRNA, and U1 RNA. The enrichment (*n*-fold) for each RNA (ZIKV, GAPDH, or U1) was calculated by comparing the amount of RNA precipitated by specific antibody (NS1, claudin-2, or HA) with the amount of the same type of RNA precipitated by isotype control IgG. The means and standard deviations from three independent experiments are shown. Statistical analysis was performed using the unpaired Student *t* test (**, *P < *0.01; NS, *P > *0.05). (C) The workflow of the *in vitro* RNA-protein pulldown assay. (D) Western blot analysis after RNA-protein pulldown assay. The GST-tagged proteins in the inputs and eluates from the pulldown assay were analyzed by Western blotting using GST antibody. (E) Competition assay. Biotinylated 5ʹ UTR-C_66_-3ʹ UTR RNA was incubated with GST-NS2A in the presence of increasing amounts of competitors. After pulldown, GST-NS2A in the eluates was detected by Western blotting (top panel). The band intensities for GST-NS2A in each lane were quantified. Relative intensity was calculated by normalizing the band intensity of each lane to that without any competitors. The means and standard deviations from three independent experiments are shown. (F) EMSA. The dissociation constant (*K_d_*) was calculated from the best-fitting curve by the least-squares fitting method. The coefficient (*R*^2^) of the curve fitting is indicated. The means and standard deviations from three independent experiments are shown.

To further explore the role of ZIKV NS2A in virion assembly, we examined the ability of NS2A to bind viral RNA using an RNA immunoprecipitation (RNA-IP) assay. WT HA-NS2A ZIKV-infected cell lysates were precipitated by antibodies against HA, viral NS1 protein, or cellular protein claudin-2 (Fig. 7A). The amount of viral RNA coprecipitated with HA-NS2A, NS1, or claudin-2 was measured by quantitative real-time RT-PCR (RT-qPCR); as controls, two cellular RNAs (GAPDH and U1) were also quantified from the precipitated samples. The enrichment of each RNA was calculated by comparison to the RNA level coprecipitated with a control IgG. As shown in Fig. 7B, about 2.5-fold enrichment of viral RNA was found in the HA-NS2A-precipitated samples, whereas no enrichments were observed for cellular GAPDH or U1 RNA. Viral NS1 or host claudin protein did not enrich ZIKV RNA, GAPDH, or U1 RNA (Fig. 7B). The results suggest a specific interaction between NS2A and viral RNA in ZIKV-infected cells.

### Specific interaction between NS2A and viral 3ʹ UTR RNA.

We performed an *in vitro* RNA-protein pulldown assay to characterize the NS2A-viral RNA interaction (Fig. 7C). Recombinant NS2A with an N-terminal glutathione *S*-transferase (GST-NS2A) was expressed from Escherichia coli, partially purified, and reconstituted in detergent *n*-Dodecyl-β-d-maltopyranoside (DDM)-containing buffer. ZIKV 5ʹ UTR-C_66_-3ʹ UTR RNA (comprising the 5ʹ UTR, the first 66 nucleotides of the C gene, and the 3ʹ UTR of viral genome) was *in vitro* transcribed and ligated to a biotinylated cytidine using T4 RNA ligase, resulting in 5ʹ UTR-C_66_-3ʹ UTR-biotin RNA. The 5ʹ UTR-C_66_-3ʹ UTR-biotin RNA efficiently pulled down GST-NS2A but not GST (Fig. 7D). In contrast, a biotinylated nonviral RNA control (an iron response element RNA) was not able to pull down GST or GST-NS2A protein (Fig. 7D). Next, we performed an RNA competition assay to demonstrate the specificity of GST-NS2A/5ʹ UTR-C_66_-3ʹ UTR RNA interaction. Increasing amounts of unbiotinylated 5ʹ UTR-C_66_-3ʹ UTR RNA competed away the GST-NS2A/5ʹ UTR-C_66_-3ʹ UTR-biotin RNA interaction, whereas yeast tRNAs did not (Fig. 7E), suggesting the specificity of the NS2A/5ʹ UTR-C_66_-3ʹ UTR RNA interaction.

### Affinity of NS2A-viral RNA interaction.

We estimated the binding affinity of the GST-NS2A/5ʹ UTR-C_66_-3ʹ UTR RNA interaction. 5ʹ UTR-C_66_-3ʹ UTR-biotin RNA was incubated with increasing concentrations of recombinant GST-NS2A protein. The reaction mixtures were separated on an agarose gel, transferred to a nylon membrane, and probed with streptavidin-horseradish peroxidase (HRP) (Fig. 7F, top panel). Quantification of free RNA and RNA/protein complex estimated a *K_d_* (dissociation constant) of 10.3 nM for ZIKV NS2A binding to viral RNA (Fig. 7F, bottom panel).

### Mapping the 3ʹ UTR responsible for NS2A binding.

**FIG 8:**
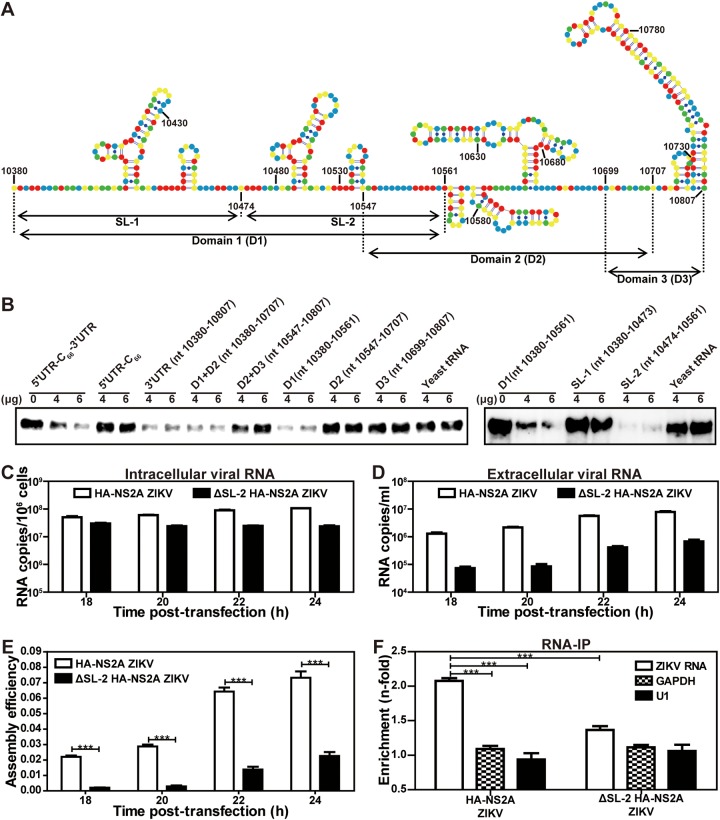
Mapping the region in ZIKV 3ʹ UTR responsible for NS2A binding. (A) The secondary structure of ZIKV 3ʹ UTR. Nucleotides A, U, C, and G are colored as blue, green, red, and yellow, respectively. RNA fragments for competition assay are indicated. (B) Mapping the regions in 5ʹ UTR-C_66_-3ʹ UTR responsible for its binding to NS2A. A competition assay was performed by adding various unbiotinylated RNA fragments to the mixture of GST-NS2A and biotinylated 5ʹ UTR-C_66_-3ʹ UTR RNA. The remaining amounts of GST-NS2A pulled down by biotinylated 5ʹ UTR-C_66_-3ʹ UTR were detected using Western blotting. (C to F) Effect of SL-2 deletion on virion assembly. An SL-2 deletion (ΔSL-2) was engineered for the HA-NS2A viral RNA. Equal amounts of WT and ΔSL-2 viral RNAs were transfected into Vero cells. At 6 h p.t., cells were washed three times with PBS and maintained in fresh medium. At 18 to 24 h p.t., quantitative RT-PCR was used to measure the intracellular (C) and extracellular (D) viral RNA levels. The ratio of extracellular to intracellular viral RNA levels was calculated to indicate virion assembly/release efficiency (E). A nonreplicative NS2A N130A RNA (22) was included as a background control (data not shown). In addition, at 24 h p.t., an RNA-IP assay was performed to measure the viral RNA binding to HA-NA2A protein (F). WT and ΔSL-2 HA-NS2A RNA-transfected cell lysates were precipitated by HA antibodies. The amount of viral RNA coprecipitated with HA-NS2A was quantified by RT-qPCR. Cellular GAPDH and U1 RNAs were included as controls.

To determine the RNA region responsible for NS2A binding, we performed a competition assay using unbiotinylated 5ʹ UTR-C_66_-3ʹ UTR RNA fragments as competitors. Figure 8A lists the RNA fragments and their locations in the 3ʹ UTR of ZIKV RNA. Unbiotinylated 3ʹ UTR RNA, not 5ʹ UTR-C_66_ RNA, competed away the GST-NS2A/biotinylated 5ʹ UTR-C_66_-3ʹ UTR interaction (Fig. 8B, left panel), suggesting that NS2A binds to the 3ʹ UTR. Since the 3ʹ UTR consists of three domains (D1, D2, and D3 [Fig. 8A]), we performed further competition assays using unbiotinylated RNA fragments representing D1, D2, D3, D1+D2, and D2+D3 as competitors. The GST-NS2A/biotinylated 5ʹ UTR-C_66_-3ʹ UTR interaction was competed away by D1 and D1+D2, not by D2, D3, or D2+D3, indicating that D1 is the main NS2A binding site (Fig. 8B, left panel). Because D1 contains two stem-loops (SL-1 and SL-2 [Fig. 8A]), we tested which stem-loop binds to NS2A. Competition results showed that, although SL-1 and SL-2 form similar secondary structures, only SL-2 efficiently competed away the GST-NS2A/biotinylated 5ʹ UTR-C_66_-3ʹ UTR interaction (Fig. 8B, right panel). Overall, the results demonstrate that ZIKV NS2A specifically binds to the SL-2 region from the 3ʹ UTR of genomic RNA.

### Amino acids R96 and R102 of NS2A are required for virion assembly.

**FIG 9:**
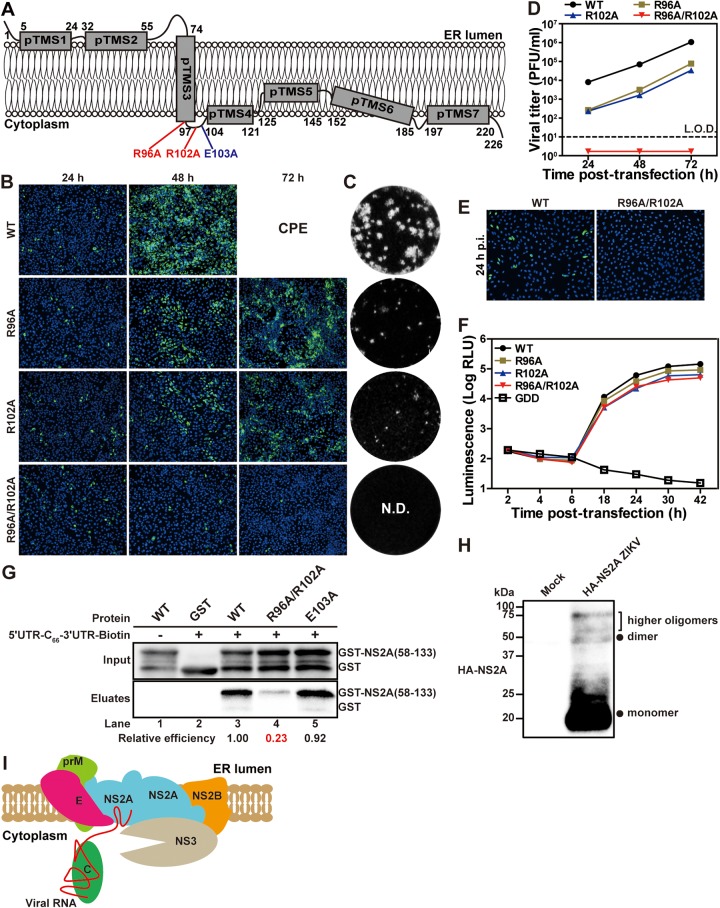
R92 and R102 residues in NS2A are essential for NS2A-viral RNA interaction and virion assembly. (A) Location of residues R96, R102, and E103 in ZIKV NS2A. (B) IFA of viral E protein expression in WT, NS2A R96A, R102A, or R96A/R102A viral RNA-transfected Vero cells. IFAs were performed at 24 to 72 h p.t. (C) Plaque morphologies of WT, R96A, and R102A ZIKVs. The NS2A R96A/R102A mutant did not yield any detectable plaques (N.D.). (D) Viral yield after transfection. The means and standard errors from three independent experiments are presented. L.O.D., limit of detection. (E) Intracellular virion infectivity. Intracellular viruses were released from the cells that were transfected with WT or R96A/R102A ZIKV RNA at 36 h p.t. by three freeze-thaw cycles. After centrifugation, the supernatants were used to infect naive Vero cells. At 24 h p.i., IFA was performed to detect E protein expression. (F) ZIKV luciferase replicon analysis. Each data point represents the mean and standard deviation from three independent experiments. (G) *In vitro* RNA-protein pulldown assay. GST-NS2A(58–133) containing amino acids 58 to 133 of NS2A and its derived mutants R96A/R102A GST-NS2A(58–133) and E103A GST-NS2A(58–133) were expressed and purified from E. coli. Equal amounts of the purified proteins were mixed with biotinylated 5ʹ UTR-C_66_-3ʹ UTR RNA in the pulldown reactions. The relative efficiency was calculated by normalizing the band intensities (as determined in the eluates) of each mutant to that of WT. (H) NS2A oligomerization in ZIKV-infected cells. Whole-cell lysates derived from HA-NS2A ZIKV-infected Vero cells or from uninfected Vero cells (mock) were analyzed by Western blotting using rabbit HA antibody. Monomer, dimer, and higher orders of oligomers are shown. (I) A model for the role of NS2A in ZIKV virion assembly. See the text for details.

Comparison of the membrane topology between ZIKV and DENV NS2A protein revealed a conserved cytoplasmic loop formed by amino acids 97 to 104 (12, 22). The cytoplasmic loop has one conserved, positively charged residue, R102; another positively charged residue, R96, located immediately upstream of the loop, is also conserved (Fig. 9). Since viral RNA is located on the cytoplasmic side of the ER membrane, we hypothesized that residues R96 and R102 might play a role in NS2A binding to viral RNA. To test this hypothesis, we prepared three recombinant ZIKVs containing Ala substitutions at single (R96A or R102A) and double (R96A/R102A) residues (Fig. 9A). After transfecting the viral RNAs into Vero cells, WT, R96A, and R102A RNAs generated increasing E-positive cells from 24 to 48 h p.t. (Fig. 9B). At 72 h p.t., about 60% and 40% of the R96A and R102A RNA-transfected cells were E-positive, respectively, whereas the WT RNA-transfected cells developed CPE (Fig. 9B). Mutants R96A and R102A produced infectious viruses with opaque plaques smaller than the WT ZIKV (Fig. 9C). At 24 to 72 h p.t., viral titers of R96A and R102A mutants were >13-fold lower than the WT (Fig. 9D). In contrast, double mutant R96A/R102A RNA did not produce any infectious virus, as indicated by no increase of E-positive cells p.t. (Fig. 9B) and no detectable extracellular viruses (Fig. 9C and D). To examine if intracellular infectious viruses were formed in the R96A/R102A RNA-transfected cells, we incubated naive Vero cells with the RNA-transfected cytosol extracts. Only the WT RNA-transfected cytosol produced E-positive Vero cells; no E-positive cells were detected after infection with the R96A/R102A RNA-transfected cytosol (Fig. 9E). These data demonstrate that single mutation R96A or R102A attenuates, and double mutation R96A/R102A abolishes, virus production.

To examine if the mutations affect viral RNA synthesis, we engineered R96A, R102A, or R96A/R102A into a luciferase ZIKV replicon. Equal amounts of WT and mutant replicon RNAs were transfected into Vero cells. At 18 to 42 h p.t., three mutant replicons produced slightly lower luciferase signals (<3-fold) than the WT (Fig. 9F), suggesting that the mutations only marginally reduce viral RNA synthesis. Together with the full-length RNA results (Fig. 9B to E), we conclude that R96 and R102 play a more important role in virion assembly than viral RNA synthesis.

### R96 and R102 are required for NS2A binding to viral RNA.

We performed an *in vitro* RNA-protein binding assay to examine the role of R96 and R102 in NS2A binding to viral RNA. To focus on the RNA binding to the NS2A cytosolic loop region, we prepared recombinant protein GST-NS2A(58–133), representing amino acids 58 to 133 of NS2A with an N-terminal GST (22). WT GST-NS2A(58–133) was efficiently pulled down by 5ʹ UTR-C_66_-3ʹ UTR-biotin RNA (Fig. 9G, lane 3), whereas double mutant R96A/R102A lost 77% of the binding to 5ʹ UTR-C_66_-3ʹ UTR-biotin (lane 4). As a negative control, E103A mutant GST-NS2A(58–133) did not compromise the RNA binding (lane 5). The results indicate that R96 and R102 are important for NS2A binding to viral RNA.

### Deletion of 3ʹ UTR SL-2 RNA (responsible for binding to NS2A) decreases virion assembly efficiency.

Figures 8B and 9 suggest that the R96/R102 loop of NS2A protein may selectively bind to the SL-2 region of 3ʹ UTR RNA during virion assembly. To demonstrate the function of SL-2 RNA in virion assembly, we deleted the SL-2 RNA region (ΔSL-2, Fig. 8A) and examined the deletion effect on virion assembly (Fig. 8C to E). After transfecting WT and ΔSL-2 HA-NS2A viral RNAs into Vero cells, RT-qPCR was performed to measure intracellular and extracellular viral RNAs. The ratio of extracellular to intracellular RNA levels was used as an indicator for virion assembly/release efficiency. A nonreplicative mutant NS2A N130A RNA (22) was included as a negative control; the intracellular and extracellular viral RNA amounts from this nonreplicative RNA served as a background for data analysis (data not shown). At 18 to 24 p.t., the WT generated <4-fold-higher intracellular viral RNAs than the ΔSL-2 mutant (Fig. 8C) but >12-fold-higher extracellular viral RNA levels (Fig. 8D), leading to significantly higher extracellular/intracellular viral RNA ratios (Fig. 8E). The result suggests that 3ʹ UTR SL-2 RNA increases the efficiency of virion assembly/release.

Next, we performed an RNA-IP assay to compare the viral RNA binding to NS2A between the WT and ΔSL-2 HA-2A RNA-transfected cells. At 24 h p.t., the cell lysates were precipitated by HA antibodies. The amount of indicated RNA coprecipitated with HA-NS2A was quantified by RT-qPCR. Compared with the cellular GAPDH and U1 RNAs, the WT viral RNA was ∼2-fold enriched in binding to HA-NS2A protein, whereas ΔSL-2 RNA was not significantly enriched (Fig. 8F). Collectively, the ΔSL-2 results provided functional evidence that the 3′ UTR SL-2 RNA region interacts with NS2A during ZIKV assembly.

## DISCUSSION

Flavivirus infection induces double-membrane vesicles (Ve) where viral replication complexes reside (23–27). At the early stage of the viral infection cycle, newly synthesized plus-sense RNAs are released from the Ve pore to the rough ER for new rounds of viral translation and replication. At the late stage of the viral infection cycle, nascent RNAs are destined for virion assembly. Virions assemble at the ER sites adjacent to Ve, where viral RNA encapsidation with C protein, envelopment with prM/E, and virion budding occur (23, 27). The mechanism of how genomic RNA and viral structural proteins are recruited to assemble virus particles is not well defined. Here, we took a combinatory genetic and biochemical approach to identify the molecular interactions that drive the process of virion assembly. Since NS2A has been well documented to participate in flavivirus virion assembly (7–14), we decided to map its interaction with other viral proteins using an unbiased co-IP approach. The co-IP results revealed that, in ZIKV-infected cells, NS2A specifically interacted with prM, E, NS2B, and NS3 (not C, NS4B, or NS5) in a viral RNA-independent manner (Fig. 3A). Corroboratively, coexpression of NS2A with prM, E, NS2B, or NS3 showed that NS2A could directly pull down each of these proteins and vice versa (Fig. 4). However, the prM/E complex does not interact with the NS2B/NS3 complex (Fig. 5). These results suggest a central role of NS2A in recruiting prM/E and NS2B/NS3 to the virion assembly site. Since DENV NS2A oligomerizes (12, 28), it is conceivable that individual prM/E and NS2B/NS3 complexes may interact with different molecules of NS2A. Indeed, we found that ZIKV NS2A could also oligomerize (Fig. 9H), and the E103A mutation did not affect the NS2A oligomerization (data not shown). Oligomerization of NS2A could bring together all the complexes for virion assembly (Fig. 9I).

The biological relevance of NS2A/prM/E and NS2A/NS2B/NS3 interactions was supported by the NS2A E103A mutation that abolished virion assembly as well as NS2A binding to prM/E or NS2B/NS3 complexes (Fig. 2 and 3A). Amino acid E103 is located at the cytoplasmic loop of NS2A. It is surprising that single-residue mutation abolished NS2A binding to all viral proteins. This mutation may change the local conformation of NS2A that is critical for those interactions. A high-resolution structure of NS2A is ultimately needed to provide the underlying mechanism. In agreement with our results, previous YFV and DENV-2 studies showed that defective virion assembly caused by NS2A mutations could be rescued by second-site mutations in prM, E, NS2B, and NS3 proteins (13, 14), providing genetic support for the NS2A/prM/E and NS2A/NS2B/NS3 interactions during flavivirus assembly. We also attempted revertant selections for the NS2A E103A mutant but failed to recover second-site mutations after several trials (data not shown).

Besides losing binding to other viral proteins, the NS2A E103A mutation also led to the production of a truncated C protein, with the first 6 amino acids removed by viral NS2B/NS3 protease (Fig. 6). We showed that the truncated C protein did not account for the defect of E103A virion assembly because the truncated protein was fully competent in producing infectious virus in the context of WT NS2A. This result suggests that, although C protein did not directly bind to NS2A, it was indirectly affected by the NS2A mutation. Since the N-terminal region of C has many positively charged residues (altogether, 6 Arg or Lys residues in the first 19 amino acids), this region may participate in genomic RNA binding and, consequently, protect the N-terminal region from protease cleavage. In the context of NS2A E103A, defective virion assembly renders the capsid N-terminal region accessible for viral protease cleavage. Overall, the result points out a functional connection between C and NS2A proteins during ZIKV assembly.

Our biochemical and functional analysis indicates that the 3ʹ UTR of viral RNA may serve as an “RNA recruitment signal” for ZIKV assembly. Although the 3ʹ UTR of Kunjin virus was reported to bind to NS2A (8), the molecular detail and biological importance have not been well characterized. Using a biotinylated RNA pulldown and competition assay, we identified a 3ʹ UTR fragment containing SL-2 as a determinant for NS2A binding (Fig. 8A and B). Since the C protein nonspecifically binds to RNA (29), the SL-2 RNA may function as a “recruitment signal” for the viral genome to be recruited for virion assembly. Functionally, deletion of SL-2 reduced the efficiency of virion assembly through a decreased viral RNA binding to NS2A protein (Fig. 8C to F). Since flavivirus replication produces abundant subgenomic flaviviral RNAs (sfRNAs), the sfRNAs represent the 3′-terminal region of viral genome and contain the SL-2 RNA element (30, 31). Future studies are needed to test if the sfRNAs might be packaged into viral particles or may modulate virion assembly. From the NS2A side, we have identified a cytoplasmic loop as the major RNA binding site. Two positively charged residues (R96 and R102) from this region are critical for RNA binding and virion assembly; substitutions of Ala for these residues reduced 77% of the RNA binding and completely abolished virus production (Fig. 9). The biological relevance of the above findings was further supported by the findings that a deletion of the 3′ UTR SL-2 RNA reduced virion assembly and viral RNA binding to NS2A (Fig. 8C to F).

During flavivirus assembly, the cleavage between C and prM is tightly regulated in a sequential manner by NS2B/NS3 protease and cellular signalase (32–34). The capsid C terminus is first cleaved by viral NS2B/NS3 on the cytoplasmic side of the ER, followed by the cleavage at the N terminus of prM by a host signalase in the ER lumen. Based on this knowledge, together with the findings in this study, we propose a model for ZIKV assembly (Fig. 9I). At a late stage of the viral infection cycle, NS2A recruits unprocessed C-prM-E, NS2B/NS3, and viral RNA to the virion assembly site. As mentioned above, these molecules could be recruited by separate NS2As and brought together through NS2A oligomerization (Fig. 9H). Once these molecules have been assembled, NS2B/NS3 protease initiates the processing of C-prM-E to produce C, prM, and E (35). The C molecules, together with those already stored at the nearby lipid droplets (36), bind to viral RNA and form nucleocapsid core, which is enveloped by prM and E proteins, leading to virion budding into the ER lumen. Although separate expressions of C, prM-E, and viral RNA could produce virus, the virus yield is significantly lower than when C-prM-E is expressed together as a single polyprotein (37–40). The model presented in Fig. 9I is also supported by our recent results for DENV assembly (41).

Finally, it should be noted that, in addition to the viral proteins described here, cellular factors (including proteins and lipids) have been well documented to participate in virion assembly (42). Studies are needed to integrate host factors into the network of flavivirus virion assembly. For instance, interactomes of NS2A and other viral proteins could be functionally examined for their roles in different steps of flavivirus infection (43, 44). Inhibition of essential protein-protein interactions will provide new avenues for antiviral development.

## MATERIALS AND METHODS

### Cell lines.

African monkey kidney epithelial (Vero E6) cells, baby hamster kidney (BHK-21) cells, and human embryo kidney 293T (HEK293T) cells were purchased from the American Type Culture Collection (ATCC) and cultured in high-glucose Dulbecco’s modified Eagle’s medium (DMEM) supplemented with 2 mM l-glutamine, 100 U/ml penicillin, 100 g/ml streptomycin, and 10% fetal bovine serum (FBS; HyClone Laboratories). BHK-NS2A-HA cells (22) constitutively expressing WT ZIKV NS2A protein with a C-terminal HA tag were maintained in DMEM supplemented with 2 mM l-glutamine, 1% penicillin-streptomycin, 10% FBS, and 750 μg/ml G418. All cells were grown at 37°C with 5% CO_2_. Medium, antibodies, and supplements were purchased from ThermoFisher Scientific (Waltham, MA).

### Antibodies.

The following antibodies were used in this study: mouse monoclonal antibody 4G2 cross-reactive with flavivirus E protein (ATCC); rabbit anti-E polyclonal antibody (GeneTex); rabbit anti-HA monoclonal antibody (Cell Signaling Technology); mouse anti-HA monoclonal antibody (Cell Signaling Technology); rabbit anti-Flag polyclonal antibody (Cell Signaling Technology); mouse anticapsid antiserum (generated in-house); rabbit anti-prM polyclonal antibody (GeneTex); rabbit anti-NS1 polyclonal antibody (GeneTex); rabbit anti-NS3 polyclonal antibody (GeneTex); mouse monoclonal antibody cross-reactive with ZIKV and DENV-2 NS4B (clone 44-4-7) (45); mouse anti-NS5 antiserum (generated in-house); mouse anti-dsRNA monoclonal antibody, clone J2 (SCICONS English & Scientific Consulting Kft); rabbit anticalnexin polyclonal antibody (Sigma-Aldrich); rabbit anti-GM130 monoclonal antibody (Cell Signaling Technology); rabbit anti-NS2B polyclonal antibody (GeneTex); rabbit anti-GAPDH polyclonal antibody (Sigma-Aldrich); rabbit anti-claudin-2 polyclonal antibody (Invitrogen); rabbit anti-GST polyclonal antibody (Abcam); goat anti-mouse IgG polyclonal antibody conjugated with Alexa Fluor 568 (ThermoFisher Scientific); goat anti-mouse IgG polyclonal antibody conjugated with Alexa Fluor 488 (ThermoFisher Scientific); goat anti-rabbit IgG polyclonal antibody conjugated with Alexa Fluor 568 (ThermoFisher Scientific); goat anti-rabbit IgG polyclonal antibody conjugated with Alexa Fluor 488 (ThermoFisher Scientific); goat anti-mouse and anti-rabbit IgG polyclonal antibodies conjugated with horseradish peroxidase (HRP) (Sigma-Aldrich); protein A conjugated with HRP (Sigma-Aldrich); rabbit IgG isotype control (Invitrogen); and mouse IgG isotype control (Invitrogen).

### Plasmid construction.

XhoI and KpnI restriction sites at positions 3018 and 5010 in the ZIKV genome were introduced into the ZIKV infectious cDNA clone (pFLZIKV) and the *Renilla* luciferase (Rluc) reporter ZIKV replicon (pZIKV Rep WT) as reported previously (22). The DNA fragments encoding the WT NS2A or NS2A mutants with an N-terminal human influenza virus hemagglutinin (HA) tag were amplified by standard overlap PCR and cloned into pFLZIKV and pZIKV Rep WT through XhoI and KpnI restriction sites, resulting in pFLZIKV HA-NS2A and pZIKV replicon HA-NS2A. To ensure the accessibility of the HA tag without interfering in the processing and function of NS2A protein, two short flexible linkers, Gly-Ser-Gly and Gly-Gly-Gly, were placed at the N terminus and C terminus of HA tag, respectively. The DNA fragments containing capsid mutations were cloned into pFLZIKV HA-NS2A through NheI and AvrII restriction sites. The DNA fragments containing 3ʹ UTR SL-2 deletion were generated by overlap PCR and inserted into pFLZIKV HA-NS2A through EcoRI and ClaI restriction sites.

A mammalian expression vector, pXJ (12), was used to construct the plasmids expressing various viral proteins and derived mutants. Constructs pXJ-NS2B-Flag and pXJ-NS3-Flag were constructed by inserting the DNA fragments encoding the NS2B or NS3 protein with a C-terminal Flag-tag sequence into the pXJ vector. pXJ-prM-Flag was constructed by cloning a DNA fragment encoding the anchor C signal peptide (C_18_) and the prM with a C-terminal Flag tag into the pXJ vector. C_18_ was retained to ensure the correct ER membrane targeting of prM. A DNA fragment encoding the last 17 amino acids of M protein (M_17_) and the E protein with a C-terminal Flag tag was cloned into pXJ vector, resulting in construct pXJ-E-Flag. M_17_ served as a signal peptide for translocation of E into the ER lumen. For the construct pXJ-HA-NS2A, a DNA fragment encoding a signal peptide from *Gaussia* luciferase (SPG), the last 16 amino acids of NS1 protein (C_16_), and NS2A with an N-terminal HA tag were fused and cloned into the pXJ vector. SPG and C_16_ sequences were engineered to ensure the correct processing and membrane topology of NS2A.

For constructs expressing full-length NS2A or its various mutants or truncations with an N-terminal glutathione *S*-transferase (GST) tag in E. coli, the corresponding DNA fragments were cloned into the vector pGEX-4T-1 (GE Healthcare).

### Recovery of recombinant viruses, plaque assay, RNA extraction, and RT-PCR.

All viral RNAs were *in vitro* transcribed from full-length cDNA clone plasmids (linearized by ClaI restriction enzyme) using the T7 mMessage mMachine kit (Ambion) and electroporated into cells (Vero or BHK-21) using the Gene Pulser XCell electroporation system (Bio-Rad) to generate virus as mentioned previously (46). Because the transfection efficiency in BHK cells is higher than that in Vero cells, we chose BHK cells when transfecting lethal mutant E103A RNA. All other mutant and WT RNAs were transfected into Vero cells. At various time points, the culture fluids from viral RNA-transfected cells were collected, filtered through a 0.22-μm polyether sulfone membrane (Millipore), and stored at −80°C. The viral titers were determined by plaque assay using a protocol described previously (22). The culture fluids were used to extract extracellular viral RNA using the QIAamp viral RNA minikit (Qiagen). The total cellular RNAs were extracted by RNeasy minikit (Qiagen). RT-PCR was performed according to the protocol described previously (22). Primers ZIKV XhoI 3009F (5ʹ-GATTATTCACTCGAGTGTGATCC-3ʹ) and ZIKV KpnI 5030R (5ʹ-GATTGGAGATCCTGAGGTACCTGCTGGGTAGT-3ʹ) were used to amplify ZIKV cDNA fragments. After 40 PCR cycles, the amplified cDNA fragments were analyzed in a 0.8% agarose gel.

### Transient replicon transfection assay.

The replicon transient-transfection assay was performed as described previously (47). Briefly, Vero cells were electroporated with Rluc-ZIKV replicon RNAs and harvested at the given time points. The luciferase activity assay was performed using the *Renilla* luciferase assay system (Promega).

### Coimmunoprecipitation assay, SDS-PAGE, and Western blotting.

Cells were lysed in IP buffer (20 mM Tris [pH 7.5], 100 mM NaCl, 0.5% DDM, and EDTA-free protease inhibitor cocktail [Roche]) with rotation at 4°C for 1 h. Cell lysates were then clarified by centrifugation at 15,000 rpm at 4°C for 15 min. The supernatants were mixed with specific antibodies or IgG isotype control (1:200 diluted), after which additional sodium chloride was added to a final concentration of 300 mM. For RNase A treatment groups, RNase A was added to a final concentration of 60 μg/ml. The mixtures were rotated overnight at 4°C to form immune complexes. Immune complexes were captured by protein A/G magnetic beads (Millipore) with 1-h rotation at 4°C. Thereafter, bead-bound immune complexes were collected by a DynaMag-2 magnet (ThermoFisher Scientific) and washed five times with chilled phosphate-buffered saline (PBS) containing 0.1% Tween 20. Finally, the bead-bound immune complexes were eluted by boiling in 2× lithium dodecyl sulfate (LDS) sample buffer supplemented with 50 mM dithiothreitol (DTT) at 70°C for 15 min. The eluates were analyzed by SDS-PAGE and Western blotting as described previously (48). Goat anti-mouse (and anti-rabbit) IgG polyclonal antibodies conjugated with HRP and protein A conjugated with HRP were used as secondary antibodies in Western blot analysis.

### Transmission electron microscopy.

Vero cells were infected with single-round infectious E103A HA-NS2A ZIKV (derived from *trans*-complementation as shown in Fig. 2E) at an MOI of 1. At 24 h p.i., cells were fixed with fixative (50 mM cacodylate buffer [pH 7.3], 2.5% formaldehyde, 0.1% glutaraldehyde, 0.01% picric acid, 0.03% CaCl_2_) for 2 h at room temperature and then washed twice with 100 mM cacodylate buffer. The monolayers were scraped off and centrifuged at 12,000 rpm for 5 min to pellet cells. The pellets were postfixed with 1% OsO_4_ in 100 mM cacodylate buffer, washed with electron microscopy (EM)-grade water, and *en bloc* stained with 2% aqueous uranyl acetate in water for 20 min at 60°C. The pellets were dehydrated in ethanol series (from 50% to 100%) and processed through propylene oxide before being embedded in Poly/Bed 812 (Polysciences). Ultrathin sections (70 nm) were cut on a Leica EM UC7 ultramicrotome (Leica Microsystems), stained with 0.4% lead citrate in water for 3 min, and examined with a Philips CM100 transmission electron microscope at 60 kV. Digital images were acquired with a bottom-mounted charge-coupled device (CCD) camera, Orius SC2001 (Gatan, Pleasanton, CA).

### RNA immunoprecipitation assay and quantitative real-time RT-PCR.

RNA immunoprecipitation (RNA-IP) was performed as described previously (49) with some modifications. Cells were lysed in lysis buffer (20 mM HEPES [pH 7.2], 150 mM KCl, 1.5 mM MgCl_2_, 0.5% DDM, 100 U/ml murine RNase inhibitor [New England Biolabs], 0.5% NP-40, 5% glycerol, and EDTA-free protease inhibitor cocktail [Roche]) with rotation for 1 h. Cell lysates were clarified by centrifugation at 13,000 rpm for 15 min, mixed with specific antibodies or IgG isotype control (1:200 dilution), and rotated overnight. Immune complexes were incubated with protein A/G magnetic beads with 1-h rotation, after which bead-bound immune complexes were washed five times with washing buffer (10 mM Tris [pH 7.5], 150 mM KCl, 5 mM MgCl_2_, 1 mM DTT, 0.1% Tween 20). All procedures for RNA-IP were completed at 4°C or on ice. Finally, buffer RLT from the RNeasy minikit (Qiagen) was added to extract the total RNA that had been captured in the bead-bound immune complexes by following the manufacturer’s instructions.

Quantitative real-time RT-PCR (RT-qPCR) was performed using an iTaq Universal SYBR Green one-step kit (Bio-Rad) on the LightCycler 480 system (Roche) by following the manufacturer’s protocol. Primers ZIKV-6878F (5ʹ-CATGGTAGCAGTGGGTCTTC-3ʹ) and ZIKV-6980R (5ʹ-CTCCTCTCTCCTTCCCATTAGA-3ʹ) were used to determine ZIKV RNA level; primers GAPDH-F (5ʹ-AGGTCGGTGTGAACGGATTTG-3ʹ) and GAPDH-R (5ʹ-TGTAGACCATGTAGTTGAGGTCA-3ʹ) were used to determine the GAPDH mRNA level; primers U1-F (5ʹ-GGGAGATACCATGATCACGAAGGT-3ʹ) and U1-R (5ʹ-ATGCAGTCGAGTTTCCCACA-3ʹ) were used to determine the U1 level. The absolute quantification of ZIKV RNA was determined by standard curve method using *in vitro*-transcribed full-length ZIKV RNAs.

### Expression and purification of recombinant ZIKV NS2A proteins in E. coli.

GST-NS2A, GST-NS2A(58–133), GST-NS2A(58–133) R96A/R102A, and GST-NS2A(58–133) E103A recombinant proteins were expressed and purified from E. coli by following a protocol as described previously (50) with modifications. In brief, pGEX-4T-1-derived plasmids encoding the corresponding GST-tagged NS2A or mutants were transformed into E. coli strain Rosetta 2(DE3)pLysS cells (Novagen). The cells were cultured in Terrific Broth medium (Invitrogen) with 100 μg/ml ampicillin and 34 μg/ml chloramphenicol at 37°C. Once the cell culture reached an optical density at 600 nm (OD_600_) of 1.0, isopropyl-β-d-thiogalactopyranoside (IPTG) was added to a final concentration of 0.5 mM to induce protein expression. After growing at 16°C overnight, the cells were pelleted down by centrifugation at 8,000 × *g* for 10 min at 4°C and resuspended in a lysis buffer (pH 7.5, 10 mM Na_2_HPO_4_, 1.8 mM KH_2_PO_4_, 2.7 mM KCl, 400 mM NaCl, 1 mM EDTA, 10 U/ml Benzonase nuclease [Millipore], and EDTA-free protease inhibitor cocktail [Roche]), followed by ultrasonication at 4°C. The lysates were clarified by centrifugation at 20,000 × *g* for 10 min. The supernatants were further centrifuged at 39,000 rpm for 1.5 h in an SW40Ti rotor (Beckman Coulter) at 4°C to pellet down the membrane fractions. Afterward, the pellet was resuspended in a solubilization buffer (pH 7.5, 10 mM Na_2_HPO_4_, 1.8 mM KH_2_PO_4_, 2.7 mM KCl, 1 M NaCl, 1 mM EDTA, 1.5% DDM) with agitation for 2 h at 4°C. After centrifugation at 39,000 rpm for 30 min to remove undissolved debris, the supernatants were then loaded onto a GSTrap FF column (GE Healthcare) preequilibrated with buffer A (pH 7.5, 10 mM Na_2_HPO_4_, 1.8 mM KH_2_PO_4_, 2.7 mM KCl, 400 mM NaCl, 0.05% DDM, 5 mM DTT, 5% glycerol). After extensive washing in buffer B (pH 7.5, 10 mM Na_2_HPO_4_, 1.8 mM KH_2_PO_4_, 2.7 mM KCl, 1 M NaCl, 0.05% DDM, 5 mM DTT, 5% glycerol), the column-bound proteins were eluted in buffer A supplemented with 20 mM glutathione. Fractions containing GST-tagged NS2A proteins were then pooled. Ultrafiltration was performed in a 50-kDa centrifugal concentrator (Sartorius) to remove the glutathione from the eluates. Finally, the eluates were concentrated to about 5 mg/ml, aliquoted, and flash-frozen in liquid nitrogen before storage at −80°C.

### RNA-protein pulldown assay.

The 5ʹ UTR-C_66_-3ʹ UTR RNAs containing the 5ʹ UTR, the first 66-nucleotide sequence of the capsid gene, and the 3ʹ UTR of the ZIKV genome were *in vitro* transcribed from a DNA template containing T7 promoter using the MEGAscript T7 transcription kit (ThermoFisher Scientific). After transcription, the reaction mixtures were passed twice through the Illustra Microspin G-25 columns (GE Healthcare) to remove ribonucleoside triphosphates (rNTPs). The RNAs were recovered by phenol-chloroform extraction and ethanol precipitation. Biotinylated 5ʹ UTR-C_66_-3ʹ UTR RNA (5ʹ UTR-C_66_-3ʹ UTR-biotin) was prepared by ligating a biotinylated cytidine (bis)phosphate to the 3ʹ end of 5ʹ UTR-C_66_-3ʹ UTR RNA using the Pierce RNA 3ʹ-end biotinylation kit (ThermoFisher Scientific) according to the manufacturer’s instructions. The biotinylated RNAs were isolated by phenol-chloroform extraction and ethanol precipitation. The biotinylated 5ʹ UTR-C_66_-3ʹ UTR RNAs were finally resuspended in RNase-free water and stored at −80°C.

RNA-protein pulldown assay was performed using the Pierce magnetic RNA-protein pulldown kit (ThermoFisher Scientific) according to the manufacturer’s instructions. Three micrograms of recombinant protein and 3 μg of biotinylated RNA were used in each pulldown reaction. After pulldown, the eluates were loaded onto SDS-PAGE gels. GST-tagged proteins in the eluates were detected by Western blotting using the rabbit anti-GST antibodies.

### EMSA.

Electrophoretic mobility shift assay (EMSA) was performed to evaluate the binding affinity of GST-NS2A to viral RNAs. In brief, 100 pM biotinylated ZIKV 5ʹ UTR-C_66_-3ʹ UTR RNAs was incubated with various concentration of recombinant GST-NS2A proteins in 50 μl binding buffer (50 mM Tris-HCl, pH 7.5, 10 mM 2-mercaptoethanol, 2 mM MgCl_2_, 50 mM KCl) for 15 min at 24°C. Afterward, 10 μl 6× gel loading dye without SDS (New England Biolabs Inc.) was added to the reaction mixtures. The mixtures were then loaded onto a 0.4% agarose gel containing 0.5× Tris-borate-EDTA (TBE) buffer to resolve the protein-RNA complexes and free RNAs by electrophoresis (120 V for 30 min) in 1× TBE buffer. Subsequently, RNAs in the agarose gel were transferred to a BrightStar-Plus positively charged nylon membrane (ThermoFisher Scientific) according to the instructions of the NorthernMax-Gly kit (Ambion). The transferred RNAs were then cross-linked to the membrane by irradiation with 200 mJ/cm^2^ of 254-nm UV light in a CX-2000 UV cross-linker (Analytik Jena). The biotinylated RNAs were detected using the chemiluminescent detection module (ThermoFisher Scientific). The chemiluminescence signals were detected using the ChemiDoc imaging systems (Bio-Rad).

The intensities of the bands representing the bound RNAs (in the protein-RNA complexes) and free RNAs in the EMSAs were quantified using the software ImageJ (NIH) to estimate the equilibrium dissociation constant (*K_d_*). The ratio of bound RNAs to the total RNAs (free plus bound RNA) versus the concentration of GST-NS2A proteins was plotted in Prism 8 (GraphPad). The value of *K_d_* was calculated using nonlinear regression with the least-squares fitting method, where the *B*_max_ was set as 1. All data show the means and standard deviations (SDs) from three independent experiments.

### Statistical analysis.

All numerical data are presented as the mean ± SD. An unpaired Student *t* test was used for statistical analysis (*, *P* < 0.05, significant; **, *P* < 0.01, very significant; ***, *P* < 0.001, highly significant; NS, *P* > 0.05, not significant).

## References

[1] Kuhn RJ, Zhang W, Rossmann MG, Pletnev SV, Corver J, Lenches E, Jones CT, Mukhopadhyay S, Chipman PR, Strauss EG, Baker TS, Strauss JH 2002 Structure of dengue virus: implications for flavivirus organization, maturation, and fusion. Cell 108:717–725. doi:10.1016/s0092-8674(02)00660-8. 11893341 PMC4152842

[2] Pierson TC, Diamond MS 2013 Flaviviruses, p 747–794. *In* Knipe DM, Howley PM, Cohen JI, Griffin DE, Lamb RA, Martin MA, Racaniello VR, Roizman B (ed), Fields virology, 6th ed, vol 1 Lippincott Williams & Wilkins, Philadelphia, PA.

[3] Khromykh AA, Varnavski AN, Sedlak PL, Westaway EG 2001 Coupling between replication and packaging of flavivirus RNA: evidence derived from the use of DNA-based full-length cDNA clones of Kunjin virus. J Virol 75:4633–4640. doi:10.1128/JVI.75.10.4633-4640.2001. 11312333 PMC114216

[4] Nugent CI, Johnson KL, Sarnow P, Kirkegaard K 1999 Functional coupling between replication and packaging of poliovirus replicon RNA. J Virol 73:427–435. 9847348 10.1128/jvi.73.1.427-435.1999 PMC103849

[5] Li L, Lok SM, Yu IM, Zhang Y, Kuhn RJ, Chen J, Rossmann MG 2008 The flavivirus precursor membrane-envelope protein complex: structure and maturation. Science 319:1830–1834. doi:10.1126/science.1153263. 18369147

[6] Yu IM, Zhang W, Holdaway HA, Li L, Kostyuchenko VA, Chipman PR, Kuhn RJ, Rossmann MG, Chen J 2008 Structure of the immature dengue virus at low pH primes proteolytic maturation. Science 319:1834–1837. doi:10.1126/science.1153264. 18369148

[7] Leung JY, Pijlman GP, Kondratieva N, Hyde J, Mackenzie JM, Khromykh AA 2008 Role of nonstructural protein NS2A in flavivirus assembly. J Virol 82:4731–4741. doi:10.1128/JVI.00002-08. 18337583 PMC2346727

[8] Mackenzie JM, Khromykh AA, Jones MK, Westaway EG 1998 Subcellular localization and some biochemical properties of the flavivirus Kunjin nonstructural proteins NS2A and NS4A. Virology 245:203–215. doi:10.1006/viro.1998.9156. 9636360

[9] Nemesio H, Villalain J 2014 Membrane interacting regions of dengue virus NS2A protein. J Phys Chem B 118:10142–10155. doi:10.1021/jp504911r. 25119664 PMC4148155

[10] Voßmann S, Wieseler J, Kerber R, Kümmerer BM 2015 A basic cluster in the N terminus of yellow fever virus NS2A contributes to infectious particle production. J Virol 89:4951–4965. doi:10.1128/JVI.03351-14. 25694595 PMC4403467

[11] Wu RH, Tsai MH, Chao DY, Yueh A 2015 Scanning mutagenesis studies reveal a potential intramolecular interaction within the C-terminal half of dengue virus NS2A involved in viral RNA replication and virus assembly and secretion. J Virol 89:4281–4295. doi:10.1128/JVI.03011-14. 25653435 PMC4442377

[12] Xie X, Gayen S, Kang C, Yuan Z, Shi PY 2013 Membrane topology and function of dengue virus NS2A protein. J Virol 87:4609–4622. doi:10.1128/JVI.02424-12. 23408612 PMC3624351

[13] Kummerer BM, Rice CM 2002 Mutations in the yellow fever virus nonstructural protein NS2A selectively block production of infectious particles. J Virol 76:4773–4784. doi:10.1128/jvi.76.10.4773-4784.2002. 11967294 PMC136122

[14] Shan C, Xie X, Zou J, Zust R, Zhang B, Ambrose R, Mackenzie J, Fink K, Shi PY 2018 Using a virion assembly-defective dengue virus as a vaccine approach. J Virol 92:e01002-18. doi:10.1128/JVI.01002-18. 30111567 PMC6189489

[15] Li XD, Deng CL, Ye HQ, Zhang HL, Zhang QY, Chen DD, Zhang PT, Shi PY, Yuan ZM, Zhang B 2016 Transmembrane domains of NS2B contribute to both viral RNA replication and particle formation in Japanese encephalitis virus. J Virol 90:5735–5749. doi:10.1128/JVI.00340-16. 27053551 PMC4886793

[16] Patkar CG, Kuhn RJ 2008 Yellow fever virus NS3 plays an essential role in virus assembly independent of its known enzymatic functions. J Virol 82:3342–3352. doi:10.1128/JVI.02447-07. 18199634 PMC2268495

[17] Johansson M, Brooks AJ, Jans DA, Vasudevan SG 2001 A small region of the dengue virus-encoded RNA-dependent RNA polymerase, NS5, confers interaction with both the nuclear transport receptor importin-beta and the viral helicase, NS3. J Gen Virol 82:735–745. doi:10.1099/0022-1317-82-4-735. 11257177

[18] Kumar A, Buhler S, Selisko B, Davidson A, Mulder K, Canard B, Miller S, Bartenschlager R 2013 Nuclear localization of dengue virus nonstructural protein 5 does not strictly correlate with efficient viral RNA replication and inhibition of type I interferon signaling. J Virol 87:4545–4557. doi:10.1128/JVI.03083-12. 23408610 PMC3624364

[19] Tay MY, Smith K, Ng IH, Chan KW, Zhao Y, Ooi EE, Lescar J, Luo D, Jans DA, Forwood JK, Vasudevan SG 2016 The C-terminal 18 amino acid region of dengue virus NS5 regulates its subcellular localization and contains a conserved arginine residue essential for infectious virus production. PLoS Pathog 12:e1005886. doi:10.1371/journal.ppat.1005886. 27622521 PMC5021334

[20] Ng IHW, Chan KW, Tan MJA, Gwee CP, Smith KM, Jeffress SJ, Saw WG, Swarbrick CMD, Watanabe S, Jans DA, Gruber G, Forwood JK, Vasudevan SG 2019 Zika virus NS5 forms supramolecular nuclear bodies that sequester importin-alpha and modulate the host immune and pro-inflammatory response in neuronal cells. ACS Infect Dis 5:932–948. doi:10.1021/acsinfecdis.8b00373. 30848123

[21] Kapoor M, Zhang L, Ramachandra M, Kusukawa J, Ebner KE, Padmanabhan R 1995 Association between NS3 and NS5 proteins of dengue virus type 2 in the putative RNA replicase is linked to differential phosphorylation of NS5. J Biol Chem 270:19100–19106. doi:10.1074/jbc.270.32.19100. 7642575

[22] Zhang X, Xie X, Zou J, Xia H, Shan C, Chen X, Shi PY 2019 Genetic and biochemical characterizations of Zika virus NS2A protein. Emerg Microbes Infect 8:585–602. doi:10.1080/22221751.2019.1598291. 30958095 PMC6455252

[23] Welsch S, Miller S, Romero-Brey I, Merz A, Bleck CK, Walther P, Fuller SD, Antony C, Krijnse-Locker J, Bartenschlager R 2009 Composition and three-dimensional architecture of the dengue virus replication and assembly sites. Cell Host Microbe 5:365–375. doi:10.1016/j.chom.2009.03.007. 19380115 PMC7103389

[24] Gillespie LK, Hoenen A, Morgan G, Mackenzie JM 2010 The endoplasmic reticulum provides the membrane platform for biogenesis of the flavivirus replication complex. J Virol 84:10438–10447. doi:10.1128/JVI.00986-10. 20686019 PMC2950591

[25] Cortese M, Goellner S, Acosta EG, Neufeldt CJ, Oleksiuk O, Lampe M, Haselmann U, Funaya C, Schieber N, Ronchi P, Schorb M, Pruunsild P, Schwab Y, Chatel-Chaix L, Ruggieri A, Bartenschlager R 2017 Ultrastructural characterization of Zika virus replication factories. Cell Rep 18:2113–2123. doi:10.1016/j.celrep.2017.02.014. 28249158 PMC5340982

[26] Miorin L, Romero-Brey I, Maiuri P, Hoppe S, Krijnse-Locker J, Bartenschlager R, Marcello A 2013 Three-dimensional architecture of tick-borne encephalitis virus replication sites and trafficking of the replicated RNA. J Virol 87:6469–6481. doi:10.1128/JVI.03456-12. 23552408 PMC3648123

[27] Junjhon J, Pennington JG, Edwards TJ, Perera R, Lanman J, Kuhn RJ 2014 Ultrastructural characterization and three-dimensional architecture of replication sites in dengue virus-infected mosquito cells. J Virol 88:4687–4697. doi:10.1128/JVI.00118-14. 24522909 PMC3993787

[28] Xie X, Zou J, Puttikhunt C, Yuan Z, Shi PY 2015 Two distinct sets of NS2A molecules are responsible for dengue virus RNA synthesis and virion assembly. J Virol 89:1298–1313. doi:10.1128/JVI.02882-14. 25392211 PMC4300643

[29] Pong WL, Huang ZS, Teoh PG, Wang CC, Wu HN 2011 RNA binding property and RNA chaperone activity of dengue virus core protein and other viral RNA-interacting proteins. FEBS Lett 585:2575–2581. doi:10.1016/j.febslet.2011.06.038. 21771593 PMC7164067

[30] Pijlman GP, Funk A, Kondratieva N, Leung J, Torres S, van der Aa L, Liu WJ, Palmenberg AC, Shi PY, Hall RA, Khromykh AA 2008 A highly structured, nuclease-resistant, noncoding RNA produced by flaviviruses is required for pathogenicity. Cell Host Microbe 4:579–591. doi:10.1016/j.chom.2008.10.007. 19064258

[31] Funk A, Truong K, Nagasaki T, Torres S, Floden N, Balmori Melian E, Edmonds J, Dong H, Shi PY, Khromykh AA 2010 RNA structures required for production of subgenomic flavivirus RNA. J Virol 84:11407–11417. doi:10.1128/JVI.01159-10. 20719943 PMC2953152

[32] Lobigs M, Lee E 2004 Inefficient signalase cleavage promotes efficient nucleocapsid incorporation into budding flavivirus membranes. J Virol 78:178–186. doi:10.1128/jvi.78.1.178-186.2004. 14671099 PMC303399

[33] Stocks CE, Lobigs M 1998 Signal peptidase cleavage at the flavivirus C-prM junction: dependence on the viral NS2B-3 protease for efficient processing requires determinants in C, the signal peptide, and prM. J Virol 72:2141–2149. doi:10.1128/JVI.74.1.24-32.2000. 9499070 PMC109509

[34] Amberg SM, Rice CM 1999 Mutagenesis of the NS2B-NS3-mediated cleavage site in the flavivirus capsid protein demonstrates a requirement for coordinated processing. J Virol 73:8083–8094. 10482557 10.1128/jvi.73.10.8083-8094.1999 PMC112824

[35] Zhang R, Miner JJ, Gorman MJ, Rausch K, Ramage H, White JP, Zuiani A, Zhang P, Fernandez E, Zhang Q, Dowd KA, Pierson TC, Cherry S, Diamond MS 2016 A CRISPR screen defines a signal peptide processing pathway required by flaviviruses. Nature 535:164–168. doi:10.1038/nature18625. 27383988 PMC4945490

[36] Samsa MM, Mondotte JA, Iglesias NG, Assuncao-Miranda I, Barbosa-Lima G, Da Poian AT, Bozza PT, Gamarnik AV 2009 Dengue virus capsid protein usurps lipid droplets for viral particle formation. PLoS Pathog 5:e1000632. doi:10.1371/journal.ppat.1000632. 19851456 PMC2760139

[37] Lobigs M, Lee E, Ng ML, Pavy M, Lobigs P 2010 A flavivirus signal peptide balances the catalytic activity of two proteases and thereby facilitates virus morphogenesis. Virology 401:80–89. doi:10.1016/j.virol.2010.02.008. 20207389

[38] Khromykh AA, Westaway EG 1997 Subgenomic replicons of the flavivirus Kunjin: construction and applications. J Virol 71:1497–1505. 8995675 10.1128/jvi.71.2.1497-1505.1997 PMC191206

[39] Harvey T, Liu W, Wang X, Linedale R, Jacobs M, Davidson A, Le T, Anraku I, Suhrbier A, Shi P, Khromykh A 2004 Tetracycline-inducible packaging cell line for production of flavivirus replicon particles. J Virol 78:531–538. doi:10.1128/jvi.78.1.531-538.2004. 14671135 PMC303381

[40] Xi X, Mo X, Xiao Y, Yin B, Lv C, Wang Y, Sun Z, Yang Q, Yao Y, Xuan Y, Li X, Yuan YA, Tian K 2016 Production of Escherichia coli-based virus-like particle vaccine against porcine circovirus type 2 challenge in piglets: structure characterization and protective efficacy validation. J Biotechnol 223:8–12. doi:10.1016/j.jbiotec.2016.02.025. 26907669

[41] Xie X, Zou J, Zhang X, Zhou Y, Routh AL, Kang C, Popov VL, Chen X, Wang QY, Dong H, Shi P-Y Dengue NS2A protein orchestrates virus assembly. Cell Host Microbe, in press. 10.1016/j.chom.2019.09.015 31631053

[42] Apte-Sengupta S, Sirohi D, Kuhn RJ 2014 Coupling of replication and assembly in flaviviruses. Curr Opin Virol 9:134–142. doi:10.1016/j.coviro.2014.09.020. 25462445 PMC4268268

[43] Shah PS, Link N, Jang GM, Sharp PP, Zhu T, Swaney DL, Johnson JR, Von Dollen J, Ramage HR, Satkamp L, Newton B, Huttenhain R, Petit MJ, Baum T, Everitt A, Laufman O, Tassetto M, Shales M, Stevenson E, Iglesias GN, Shokat L, Tripathi S, Balasubramaniam V, Webb LG, Aguirre S, Willsey AJ, Garcia-Sastre A, Pollard KS, Cherry S, Gamarnik AV, Marazzi I, Taunton J, Fernandez-Sesma A, Bellen HJ, Andino R, Krogan NJ 2018 Comparative flavivirus-host protein interaction mapping reveals mechanisms of dengue and Zika virus pathogenesis. Cell 175:1931–1945.e1918. doi:10.1016/j.cell.2018.11.028. 30550790 PMC6474419

[44] Scaturro P, Stukalov A, Haas DA, Cortese M, Draganova K, Płaszczyca A, Bartenschlager R, Götz M, Pichlmair A 2018 An orthogonal proteomic survey uncovers novel Zika virus host factors. Nature 561:253–257. doi:10.1038/s41586-018-0484-5. 30177828

[45] Xie X, Zou J, Wang QY, Noble CG, Lescar J, Shi PY 2014 Generation and characterization of mouse monoclonal antibodies against NS4B protein of dengue virus. Virology 450–451:250–257. doi:10.1016/j.virol.2013.12.025. 24503088

[46] Xie X, Wang QY, Xu HY, Qing M, Kramer L, Yuan Z, Shi PY 2011 Inhibition of dengue virus by targeting viral NS4B protein. J Virol 85:11183–11195. doi:10.1128/JVI.05468-11. 21865382 PMC3194949

[47] Xie X, Zou J, Shan C, Yang Y, Kum DB, Dallmeier K, Neyts J, Shi PY 2016 Zika virus replicons for drug discovery. EBioMedicine 12:156–160. doi:10.1016/j.ebiom.2016.09.013. 27658737 PMC5078599

[48] Yang Y, Shan C, Zou J, Muruato AE, Bruno DN, de Almeida Medeiros Daniele B, Vasconcelos PFC, Rossi SL, Weaver SC, Xie X, Shi PY 2017 A cDNA clone-launched platform for high-yield production of inactivated Zika vaccine. EBioMedicine 17:145–156. doi:10.1016/j.ebiom.2017.02.003. 28196656 PMC5360567

[49] Phillips SL, Soderblom EJ, Bradrick SS, Garcia-Blanco MA 2016 Identification of proteins bound to dengue viral RNA in vivo reveals new host proteins important for virus replication. mBio 7:e01865-15. doi:10.1128/mBio.01865-15. 26733069 PMC4725007

[50] Zou J, Xie X, Lee LT, Chandrasekaran R, Reynaud A, Yap L, Wang Q-Y, Dong H, Kang C, Yuan Z, Lescar J, Shi P-Y 2014 Dimerization of flavivirus NS4B protein. J Virol 88:3379–3391. doi:10.1128/JVI.02782-13. 24390334 PMC3957939

